# Risk factors for extubation-related complications in morbidly obese patients undergoing bariatric surgery: a retrospective cohort study

**DOI:** 10.1007/s00540-025-03484-z

**Published:** 2025-03-28

**Authors:** Ayten Saracoglu, Atchyuta R. R. Vegesna, Bushra M. Abdallah, Al Muiz Osama Ahmed Idrous, Amgad M. Elshoeibi, Cecil Ninan Varghese, Osman Osama Elhassan, Afrin Shakeel, Mohsen Karam, Mohammed Rizwan, Moataz M. Bashah, Kemal T. Saracoglu

**Affiliations:** 1 Department of Anesthesiology, ICU and Perioperative Medicine, Hamad Medical Corporation, Doha, Qatar; 2 College of Medicine, QU Health, Qatar University, P O BOX 2713, Doha, Qatar; 3 Department of Medical Education, Hamad Medical Corporation, Doha, Qatar; 4 Division of Bariatric and Metabolic Surgery, Department of Surgery, Hamad Medical Corporation, Doha, Qatar

**Keywords:** Airway management, Bariatric surgery, Extubation complications, Morbid obesity

## Abstract

**Background:**

One-third of major anesthesia-related airway complications occur during or shortly after tracheal extubation. Obesity significantly impacts respiratory function and is a key contributor to morbidity and mortality. Patients with morbid obesity often require bariatric surgery. However, extubation-related complications in this specific surgical population have not been previously studied. This study aimed to determine the rate and frequency of complications during tracheal extubation in patients undergoing bariatric surgery and the associated risk factors for these complications.

**Materials and methods:**

This was a retrospective cohort study of adult patients above 18 years of age with a body mass index ≥ 40 kg/m^2^ who underwent bariatric surgery between June 2016 and June 2024. Extubation-related complications were defined as the occurrence of any of the following: vomiting, aspiration, laryngospasm, bronchospasm, cardiovascular instability, airway edema, desaturation (SpO_2_ < 90%), or the need for a rescue device or reintubation during or after tracheal extubation. Logistic regression analysis, adjusted for age and sex, was performed to evaluate the associations. The significance level was adjusted by applying the Bonferroni correction (0.05/16 = 0.0031), and a *p*-value < 0.0031 was interpreted as statistically significant.

**Results:**

Data from 1193 patients were analyzed. The overall complication rate was 4.4%, with the most frequent complication being desaturation, which occurred in 3.2% of patients. Logistic regression analysis showed that the odds of extubation-related complications increased twofold for obese patients with body mass index 50–59 kg/m^2^ (odds ratio [OR] 1.97, 95% confidence interval [95%CI] 0.99–3.94, *p* = 0.055) and threefold for patients with body mass index > 60 kg/m^2^ (OR 2.95, 95%CI 0.99–8.81, *p* = 0.05). The most commonly associated comorbidities were hypertension and obstructive sleep apnea, with an odds ratio of 2.98 for hypertension and 2.15 for obstructive sleep apnea (95%CI 1.40–6.33, *p* = 0.005; and 95%CI 1.08–4.29, *p* = 0.03; respectively). Despite these clinically important results, after applying the Bonferroni correction, none of these associations remain statistically significant, as the corrected p-values are above the threshold of *p* = 0.0031.

**Conclusion:**

This study identified desaturation as the most common complication post-extubation of morbidly obese patients who underwent bariatric surgery. Moreover, we found that the odds of extubation-related complications increased with increasing obesity classes, particularly in patients with body mass index 50–59 kg/m^2^ and > 60 kg/m^2^, as well as in patients with hypertension and obstructive sleep apnea. These findings suggest the importance of tailored extubation strategies and close perioperative monitoring in morbidly obese patients to mitigate extubation-related risks.

## Introduction

Morbid obesity, defined as a body mass index ≥ 40 kg/m^2^, is associated with various comorbidities, such as obstructive sleep apnea, gastroesophageal reflux disease, hypertension, and asthma, all of which complicate airway management [1–3]. Patients with morbid obesity are especially susceptible to challenges in airway management such as airway obstruction, difficult mask ventilation, and difficult intubation, primarily due to excessive adipose tissue around the neck and upper airway, which narrows the pharyngeal lumen and restricts head movement [4].

The incidence of extubation complications in morbidly obese bariatric patients is an area with limited previous research. Most studies focus on broader perioperative respiratory risks or intubation-related outcomes rather than extubation outcomes [5, 6]. Several knowledge gaps remain, including the incidence and predictors of extubation failure, the role of airway management strategies, post-extubation respiratory complications, and long-term outcomes after extubation. Difficulties such as mask ventilation, securing the airway, and visualizing anatomical structures during intubation have been extensively studied, with a particular focus on video laryngoscopy and other intubation aids [1, 7]. However, the perioperative period extends beyond intubation, and extubation-related risks remain underexplored despite being equally critical to patient safety.

Earlier studies reported that increasing body mass index in obese patients is associated with significant morbidity and mortality, requiring greater hospital resources and expenses [8–10]. Moreover, a study investigating the incidence of postoperative pulmonary atelectasis reported a greater rate of atelectasis in obese patients than in nonobese patients following tracheal extubation (7.6% vs. 2.8%; *p* < 0.05) [11]. Additionally, multiple studies have demonstrated that complications during and after extubation occur more frequently than those during induction of anesthesia [12, 13]. However, extubation-specific data in morbidly obese patients remain scarce, and the effectiveness of airway management strategies, including specific extubation protocols and adjunct devices, has not been thoroughly investigated. Recognizing these gaps, we identified the need for a large cohort study on extubation-related complications in morbidly obese patients, as such findings could provide valuable insights to help clinicians enhance patient safety.

Existing studies present conflicting data on extubation failure rates in obese patients. Juang et al. [14] reported that 13% of adult non-obese patients experienced at least one respiratory complication during deep extubation. Ilyas et al. [15] concluded that the incidence of failed extubation significantly increased when the mean body mass index reached 33.5 ± 3.1. Conversely, a prospective multicenter observational study analyzing 1370 extubation procedures found no significant difference in failure rates between obese and non-obese patients, reporting incidences of 8% and 11%, respectively (unadjusted OR: 0.71; *P* = 0.15) [16].

Given the increasing prevalence of morbid obesity worldwide and the increasing number of bariatric surgeries performed, understanding extubation-related complications in this population is critical for improving perioperative safety [17–22]. Addressing this knowledge gap would provide insights into tailored airway management strategies, improve perioperative protocols, minimize extubation-related complications, and enhance patient outcomes in morbidly obese individuals undergoing bariatric surgery. As bariatric surgery has become the primary intervention for weight loss in morbidly obese patients, ensuring safe perioperative airway management is of significant importance. This study, therefore, aimed to evaluate the frequency and types of extubation-related complications in morbidly obese patients who underwent bariatric surgery and identify the risk factors associated with their development.

## Methods

### Study design and participants

This was a retrospective cohort study that adhered to the STROBE Statement for cohort studies and received institutional review board approval (MRC-01–24-244). This study was performed between May 2024 and November 2024 and used data obtained from the electronic medical records of all eligible patients with morbid obesity who underwent bariatric surgery at Hamad Medical Corporation between June 2016 and June 2024.

Written informed consent was not required for this study due to its retrospective nature. However, as per our institutional policies, patient data confidentiality and anonymity were strictly maintained. The data collection was performed under the Ministry of Public Health guidelines category 3a, which states that research involving the collection or study of existing data, documents, records and the information shall be recorded by the investigators in such a manner that subjects cannot be identified, directly or through identifiers linked to the subjects.

All adult patients with morbid obesity, defined as a body mass index of ≥ 40 kg/m^2^ [23] and above 18 years of age, who underwent bariatric surgery under general anesthesia in the operating theaters of Hamad Medical Corporation between June 2016 and June 2024 were included. Excluded individuals were pediatric patients younger than 18 years, adult patients with a body mass index < 40 kg/m^2^, those who had surgeries other than bariatric surgery, or those who had sedation, regional anesthesia, or local anesthesia.

### Data collection and variables

At the Cerner® login window of the electronic medical records system, anesthetists at our institution used their personal usernames and passwords to access the computer designated for operating rooms. They searched for patients' healthcare ID numbers to locate and document intraoperative anesthesia records, including medications administered and any complications that occurred. Vital signs were automatically recorded in the system during the perioperative and intraoperative periods. For this study, the authors extracted data from the electronic medical records of the study participants via a standardized data collection sheet, pre-prepared in Microsoft Excel specifically for the study.

We collected data on 1) participant characteristics such as age, sex, height, weight, and medical history; 2) preoperative anesthesia assessment, including American Society of Anesthesiologists (ASA) physical status classification, Mallampati score, Cormack-Lehane grade, and measurements of thyromental distance and mouth opening; 3) intraoperative airway management and anesthesia data; 4) postoperative data on extubation-related complications, including vomiting, aspiration, laryngospasm, bronchospasm, cardiovascular instability (defined as 20% change from baseline heart rate or blood pressure), airway edema, desaturation (SpO_2_ < 90%), or the need for a rescue device or reintubation during or after extubation; and 5) admission data, including post-anesthesia care unit stay duration, intensive care unit stay duration, and hospital stay duration.

According to our institutional protocols, patients underwent intravenous induction with propofol and fentanyl. For the induction of general anesthesia, fentanyl (2 mcg/kg), lidocaine (0.5 mg/kg), propofol (2 mg/kg), and rocuronium (1 mg/kg) were administered. Anesthesia was maintained with sevoflurane, targeting an age-adjusted minimal alveolar concentration of 1.0 in a mixture of air and oxygen with an inspired oxygen fraction of 40%—50%. The sevoflurane concentration was adjusted to maintain a Bispectral Index level between 40 and 60.

The participants were classified into three categories based on their body mass index measurement. Participants with a body mass index ≥ 40 kg/m^2^ and < 50 kg/m^2^ were categorized as class 3 obesity, participants with a body mass index ≥ 50 kg/m^2^ and < 60 kg/m^2^ were categorized as class 4 obesity, and participants with a body mass index ≥ 60 kg/m^2^ were categorized as class 5 obesity [23, 24].

### Statistical analysis

Continuous variables were assessed for normality via the Shapiro–Wilk test and histograms. Non-normally distributed continuous variables were summarized as medians and interquartile ranges, while categorical variables were summarized as frequencies and percentages. Kruskal–Wallis test was used to test the differences between skewed continuous variables, and Chi-square test was used for categorical variables.

Multivariable logistic regression was employed to assess the associations between potential predictors and risk factors and the development of extubation-related complications. We ran a total of 16 multivariable logistic regression models, once for each independent variable being analyzed. Fifteen of these models were adjusted for age and sex, whereas the model analyzing the sex variable was adjusted for age only. Odds ratios (ORs), their 95% confidence intervals (95% CIs), and the exact *p* values were reported. To minimize the risk of false positives associated with running multiple comparisons, we have adjusted the significance level by applying the Bonferroni correction (0.05/16 = 0.0031), and a *p*-value < 0.0031 was interpreted as statistically significant. All the statistical analyses were carried out via Stata 17.0 (StataCorp, College Station, TX, USA).

## Results

### Baseline characteristics

This study included a total of 1193 adults with morbid obesity who underwent bariatric surgery. Of these, 956 had class 3 obesity, 193 had class 4 obesity, and 44 had class 5 obesity. Table 1 outlines the baseline characteristics of these patients. Patients in class 5 had the highest median age (36 years) compared to class 3 (33 years) and class 4 (31 years) patients. The most commonly performed procedure was laparoscopic sleeve gastrectomy. Patients in class 3 had the shortest median duration of stay in the post-anesthesia care unit (70 min). Similarly, class 3 had the smallest proportion of patients requiring admission to intensive care (2.6%) compared with class 4 and class 5 patients (6.7% and 6.8%, respectively). Table 2 summarizes the preoperative and intraoperative anesthesia assessment characteristics of the participants.

**Table 1:** Characteristics of the participants

Variable	Level	Class 3 obesity	Class 4 obesity	Class 5 obesity
N		956	193	44
Age, median (IQR)		33 (24, 42)	31 (21, 43)	36 (22, 46)
Sex	Female	644 (67.4%)	110 (57.0%)	27 (61.4%)
	Male	312 (32.6%)	83 (43.0%)	17 (38.6%)
Height (m), median (IQR)		1.6 (1.6, 1.7)	1.6 (1.6, 1.7)	1.6 (1.5, 1.7)
Body Weight (kg), median (IQR)		115 (106, 128)	145 (129, 161)	165 (151, 182)
Comorbidities	No	673 (70.4%)	118 (61.1%)	24 (54.5%)
	Yes	283 (29.6%)	75 (38.9%)	20 (45.5%)
Number of Comorbidities	0	673 (70.4%)	118 (61.1%)	24 (54.5%)
	1	67 (7.0%)	17 (8.8%)	4 (9.1%)
	2	129 (13.5%)	27 (14.0%)	8 (18.2%)
	3	74 (7.7%)	26 (13.5%)	5 (11.4%)
	4	13 (1.4%)	5 (2.6%)	3 (6.8%)
Asthma	No	882 (92.3%)	163 (84.5%)	36 (81.8%)
	Yes	74 (7.7%)	30 (15.5%)	8 (18.2%)
Gastroesophageal Reflux Disease	No	917 (95.9%)	188 (97.4%)	43 (97.7%)
	Yes	39 (4.1%)	5 (2.6%)	1 (2.3%)
Hypertension	No	823 (86.1%)	155 (80.3%)	33 (75.0%)
	Yes	133 (13.9%)	38 (19.7%)	11 (25.0%)
Diabetes Mellitus	No	815 (85.3%)	154 (79.8%)	33 (75.0%)
	Yes	141 (14.7%)	39 (20.2%)	11 (25.0%)
Obstructive Sleep Apnea	No	744 (77.8%)	136 (70.5%)	28 (63.6%)
	Yes	212 (22.2%)	57 (29.5%)	16 (36.4%)
Procedure	LRYGB	32 (3.3%)	6 (3.1%)	1 (2.3%)
	LSG	856 (89.5%)	174 (90.2%)	41 (93.2%)
	LSG to LRYGB	30 (3.1%)	3 (1.6%)	0 (0.0%)
	LSG to MGB	18 (1.9%)	2 (1.0%)	0 (0.0%)
	MSG	18 (1.9%)	8 (4.1%)	2 (4.5%)
	MSG to LRYGB	2 (0.2%)	0 (0.0%)	0 (0.0%)
PACU Stay (mins), median (IQR)		70 (60, 90)	75 (60, 110)	75 (60, 105)
Hospital Stay (days), median (IQR)		2 (2, 3)	2 (2, 3)	3 (2, 3)
ICU Admission	No	931 (97.4%)	180 (93.3%)	41 (93.2%)
	Yes	25 (2.6%)	13 (6.7%)	3 (6.8%)
Duration of ICU Stay (days), median (IQR)		1 (1, 2)	2 (1, 3)	2 (1, 2)

*PACU* postanesthesia care unit, *ICU* intensive care unit, *IQR* interquartile range, *LRYGB* laparoscopic Roux-en-Y gastric bypass, *LSG* laparoscopic sleeve gastrectomy, *MSG* modified sleeve gastrectomy

**Table 2:** Preoperative and intraoperative anesthesia characteristics

Variable	Level	Class 3 obesity	Class 4 obesity	Class 5 obesity
N		956	193	44
ASA score	1	11 (1.2%)	1 (0.5%)	0 (0.0%)
	2	697 (72.9%)	101 (52.3%)	15 (34.1%)
	3	228 (23.8%)	86 (44.6%)	26 (59.1%)
	4	2 (0.2%)	2 (1.0%)	2 (4.5%)
	NR	18 (1.9%)	3 (1.6%)	1 (2.3%)
Mallampati score	I	133 (13.9%)	17 (8.8%)	2 (4.5%)
	II	607 (63.5%)	123 (63.7%)	28 (63.6%)
	III	131 (13.7%)	34 (17.6%)	10 (22.7%)
	IV	10 (1.0%)	4 (2.1%)	1 (2.3%)
	NR	75 (7.8%)	15 (7.8%)	3 (6.8%)
Cormack-Lehane Grade	I	210 (22.0%)	54 (28.0%)	11 (25.0%)
	II	408 (42.7%)	76 (39.4%)	12 (27.3%)
	III	47 (4.9%)	7 (3.6%)	1 (2.3%)
	IIa	104 (10.9%)	22 (11.4%)	1 (2.3%)
	IIb	23 (2.4%)	6 (3.1%)	1 (2.3%)
	IV	6 (0.6%)	0 (0.0%)	1 (2.3%)
	NR	158 (16.5%)	28 (14.5%)	17 (38.6%)
STOP-Bang score	Low Risk	79 (8.3%)	18 (9.3%)	3 (6.8%)
	Intermediate Risk	83 (8.7%)	18 (9.3%)	2 (4.5%)
	High Risk	20 (2.1%)	9 (4.7%)	3 (6.8%)
	NR	774 (81.0%)	148 (76.7%)	36 (81.8%)
Short Neck	No	940 (98.3%)	190 (98.4%)	41 (93.2%)
	Yes	16 (1.7%)	3 (1.6%)	3 (6.8%)
Mouth Opening (cm), median (IQR)		4.0 (3.0, 5.0)	4.0 (3.5, 5.0)	5.0 (4.0, 5.0)
Thyromental Distance (cm)	< 6	282 (29.5%)	49 (25.4%)	14 (31.8%)
	6 to 6.5	348 (36.4%)	75 (38.9%)	15 (34.1%)
	> 6.5	62 (6.5%)	18 (9.3%)	4 (9.1%)
	NR	264 (27.6%)	51 (26.4%)	11 (25.0%)
Number of Intubation Attempts	1	868 (90.8%)	180 (93.3%)	40 (90.9%)
	2	37 (3.9%)	6 (3.1%)	1 (2.3%)
	3	4 (0.4%)	1 (0.5%)	0 (0.0%)
	4	1 (0.1%)	0 (0.0%)	0 (0.0%)
	NR	46 (4.8%)	6 (3.1%)	3 (6.8%)

*ASA* American society of anesthesiologists, *IQR* interquartile range, *NR* not reported

### Incidence of extubation-related complications

A total of 53 extubation-related complications were observed among 47 participants (Table 3). Two of these participants experienced two complications each, whereas another two participants experienced three complications each. The majority of complications were attributed to desaturation following extubation (*N* = 38). Compared to class 4 and class 3 patients (5.2% and 2.5%, respectively), class 5 patients had the highest proportion of patients with desaturation post-extubation (9.1%). Reintubation and cardiovascular instability each occurred five times, whereas the need for a rescue device, vomiting or aspiration, and airway edema during extubation were observed once each (0.1%). Among the two participants with two complications, one required reintubation and experienced airway edema, whereas the other required reintubation and experienced desaturation. The two participants with three complications each experienced more severe events: one participant developed cardiovascular instability, required a rescue device during extubation, and needed reintubation; the other experienced laryngospasm/bronchospasm, desaturation during extubation, and required reintubation.

**Table 3:** The type of complication following extubation

Variable	Level	Class 3 obesity	Class 4 obesity	Class 5 obesity
N		956	193	44
Need for Rescue Device in Extubation	No	955 (99.9%)	193 (100.0%)	44 (100.0%)
	Yes	1 (0.1%)	0 (0.0%)	0 (0.0%)
Vomiting or Aspiration	No	955 (99.9%)	193 (100.0%)	44 (100.0%)
	Yes	1 (0.1%)	0 (0.0%)	0 (0.0%)
Airway Edema	No	956 (100.0%)	192 (99.5%)	44 (100.0%)
	Yes	0 (0.0%)	1 (0.5%)	0 (0.0%)
Laryngospasm or Bronchospasm	No	954 (99.8%)	193 (100.0%)	44 (100.0%)
	Yes	2 (0.2%)	0 (0.0%)	0 (0.0%)
Reintubation	No	953 (99.7%)	191 (99.0%)	44 (100.0%)
	Yes	3 (0.3%)	2 (1.0%)	0 (0.0%)
Cardiovascular Instability	No	951 (99.5%)	193 (100.0%)	44 (100.0%)
	Yes	5 (0.5%)	0 (0.0%)	0 (0.0%)
Desaturation of <90% after Extubation	No	932 (97.5%)	183 (94.8%)	40 (90.9%)
	Yes	24 (2.5%)	10 (5.2%)	4 (9.1%)

### Logistic regression analysis

Table 4 presents the logistic regression analysis, which showed that male sex was associated with a twofold increase in the odds of developing extubation-related complications (OR 2.02, 95%CI 1.11–3.67, *p* = 0.02). Similarly, class 4 obesity and class 5 obesity increased the odds by twofold and threefold, respectively (class 4 obesity, OR 1.97, 95%CI 0.99–3.94, *p* = 0.055; class 5 obesity, OR 2.95, 95%CI 0.99–8.81, *p* = 0.05), although the lower limit of the 95% CIs suggests that this association should be interpreted with caution. Having comorbidities increased the odds of extubation-related complications by 1.83 times (OR 1.83, 95%CI 0.96–3.48, *p* = 0.07), although this association lacked statistical significance. The most commonly associated comorbidities were hypertension and obstructive sleep apnea, with an OR of 2.98 for hypertension (95%CI 1.40–6.33, *p* = 0.005) and an OR of 2.15 for obstructive sleep apnea (95%CI 1.08–4.29, *p* = 0.03). Despite these clinically important results, after applying the Bonferroni correction (0.05/16 = 0.0031), none of these associations remain statistically significant, as the corrected p-values are above the threshold of *p* = 0.0031.

**Table 4:** Logistic regression of the association between the independent variables and the development of difficult extubation

Variable	Levels	Odds ratio	95% CI	p-value	Reference
Sex	Male	2.02	1.11 – 3.67	0.02	Female
Body Mass Index	Class 4 Obesity	1.97	0.99 – 3.94	0.055	Class 3 Obesity
	Class 5 Obesity	2.95	0.99 – 8.81	0.05	Class 3 Obesity
ASA Physical Status Classification	ASA 3	1.20	0.62 – 2.33	0.58	ASA 2
	ASA 4	4.23	0.44 – 40.7	0.21	ASA 2
Comorbidity	Yes	1.83	0.96 – 3.48	0.07	No
Number of Comorbidities	1	1.15	0.34 – 3.89	0.83	0
	2	1.67	0.71 – 3.91	0.24	0
	3	3.16	1.27 – 7.82	0.01	0
	4	1.78	0.21 – 14.7	0.59	0
Diabetes Mellitus	Yes	1.42	0.66 – 3.09	0.37	No
Asthma	Yes	1.20	0.46 – 3.11	0.71	No
Hypertension	Yes	2.98	1.40 – 6.33	0.005	No
Obstructive Sleep Apnea	Yes	2.15	1.08 – 4.29	0.03	No
Mallampati Score	MS II	0.88	0.35 – 2.16	0.77	MS I
	MS III	0.89	0.29 – 2.74	0.84	MS I
	MS IV	1.16	0.12 – 10.9	0.90	MS I
Cormack–Lehane Grade	CLG II	0.68	0.31 – 1.53	0.36	CLG I
	CLG IIa	0.38	0.08 – 1.73	0.21	CLG I
	CLG IIb	0.77	0.10 – 6.18	0.80	CLG I
	CLG III	1.54	0.45 – 5.30	0.49	CLG I
	CLG IV	2.90	0.29 – 28.9	0.36	CLG I
Thyromental Distance (cm)	6 to 6.5	0.76	0.41 – 1.41	0.38	< 6
	> 6.5	0.15	0.02 – 1.14	0.07	< 6
Tracheal Tube Size	7.5	1.43	0.71 – 2.89	0.32	7
	8	2.12	0.74 – 6.07	0.16	7
Tracheal Tube Cuff Pressure (cmH_2_O)	Ideal (20–30)	5.13	0.69 – 38.0	0.11	< 20
Laryngoscope Blade Number	3.5	4.38	0.47 – 41.0	0.20	3
	4	0.76	0.36 – 1.57	0.45	3
Number of Intubation Attempts	> 1	0.94	0.22 – 4.05	0.93	1

*95% CI* 95% confidence interval, *ASA* American society of anesthesiologists, *MS* Mallampati score, *CLG* Cormack–Lehane grade

## Discussion

This study explored extubation-related complications in 1193 adults with morbid obesity who underwent bariatric surgery. Logistic regression analysis identified male sex, class 4 obesity, class 5 obesity, hypertension, and obstructive sleep apnea were potential risk factors for extubation-related complications. Male patients had nearly double the odds of complications, whereas class 5 obesity tripled the odds. Hypertension and obstructive sleep apnea were also associated with increased odds of post-extubation complications, with ORs of 2.98 and 2.15, respectively.

The overall extubation complication rate observed in this study was 4.4%, with desaturation (SpO₂ < 90%) being the most common complication (3.2%). These rates are lower than those reported in previous literature, which often highlights higher incidences of perioperative respiratory complications in obese patients, particularly due to restrictive pulmonary physiology and comorbidities such as obstructive sleep apnea [25, 26]. Several factors may explain this discrepancy. The study was conducted at a single center with a dedicated bariatric surgery program, where experienced anesthesiologists and perioperative teams, who are extensively trained in the management of obese patients, likely contributed to reduced variability in care and minimized complications. Standardized protocols, including comprehensive preoperative assessments for comorbidities such as obstructive sleep apnea and hypertension, as well as consistent intraoperative airway management strategies, may have further mitigated risks compared with multicenter studies or those conducted in less specialized settings. Additionally, the retrospective design and the specific definition of extubation-related complications used in this study may have influenced the lower reported incidence. The strict inclusion criteria, which limited the study to bariatric surgery patients and excluded nonobese or non-bariatric populations, may also account for differences in complication rates compared with broader studies. Therefore, comparisons with previous research should be interpreted with these contextual differences in mind.

In our study, higher obesity classes, particularly class 4 and class 5, were associated with an increased likelihood of extubation-related complications, although the confidence intervals overlapped slightly due to sample size limitations in these groups. Specifically, patients in class 4 and class 5 obesity were two and three times more likely, respectively, to experience complications. Moreover, elevated body mass index exacerbates difficulties by adding pressure on the thoracic cavity and reducing lung compliance, increasing the risk of desaturation [27]. This was the most frequent extubation complication observed in the current study, with a rate of 3.2%.

Comorbidities, especially hypertension and obstructive sleep apnea, were found to be predictors of extubation complications. Hypertension increased the odds of extubation-related complications by threefold, whereas obstructive sleep apnea increased the odds by twofold. Hypertension and obstructive sleep apnea are independently associated with increased risks of airway complications, as they both contribute to airway resistance and cardiovascular instability [28, 29]. Patients with obstructive sleep apnea are particularly vulnerable due to airway collapsibility, which can lead to hypoxemia during extubation, emphasizing the need for comprehensive preoperative evaluation and careful respiratory monitoring in this group [30].

Compared with female patients, male patients were almost twice as likely to experience extubation complications in our study. This sex difference may be due to higher rates of obstructive sleep apnea and increased neck circumference in men, both of which are factors known to complicate airway management. These characteristics contribute to greater airway obstruction risk, particularly post-extubation. Although few studies have directly addressed sex differences in extubation outcomes, our findings align with the broader literature indicating increased intubation and extubation challenges in male patients with obstructive sleep apnea. Thille et al. [31] reported a significantly higher rate of reintubation at 48 h in males in the intensive care unit.

This study highlights the increased odds of extubation-related complications in patients with higher classes of obesity. These findings support the need for careful preoperative evaluation and consideration of tailored perioperative management strategies for patients with morbid obesity and associated comorbidities. Suggested strategies include the use of non-invasive ventilation or high-flow nasal cannula oxygenation in the immediate postoperative period for patients with severe obesity and obstructive sleep apnea. Non-invasive ventilation has been shown to reduce the risk of postoperative respiratory failure in high-risk populations [5]. In a meta-analysis of 20 randomized controlled trials involving 1184 obese patients, high-flow nasal cannula oxygenation was found to be the optimal choice to decrease the risk of postoperative pulmonary complications compared with non-invasive ventilation [32]. Additionally, close monitoring of oxygenation and ventilation immediately following extubation is crucial to identify early signs of hypoxemia or respiratory distress, particularly in patients with known risk factors. Advanced techniques for extubation can also be considered for patients with morbid obesity [33].

Extubation is a critical step in the perioperative process, marking the transition from mechanical ventilation to independent spontaneous respiration. The choice of perioperative anesthesia agent plays a critical role in the success rate of extubation. Balancing the pharmacological effects of these agents with patient-specific needs and employing evidence-based practices can optimize extubation outcomes and reduce the complication rate. An individualized approach that considers the pharmacodynamics and pharmacokinetics of anesthetic agents, along with patient-specific factors can improve overall surgical recovery. Further research and the integration of advanced monitoring tools can enhance decision-making and patient safety in this context.

There are several potential limitations to this study. First, we conducted this study at a single medical facility, so the findings may lack generalizability. Second, no comparisons were made between morbidly obese patients and nonobese patients, or between morbidly obese patients undergoing bariatric surgery and other types of surgeries. Finally, as the study was retrospective, incomplete data, unmeasured confounders, and the use of interventions such as high-flow nasal cannula oxygenation or non-invasive ventilation might differ across patients, which could introduce variability in outcomes and limit consistency across cases.

## Conclusion

This study identified desaturation as the most common complication post-extubation in morbidly obese patients undergoing bariatric surgery. Moreover, we found that the odds of extubation-related complications increased with increasing obesity classes, particularly in class 4 and class 5 obesity, as well as in patients with hypertension and obstructive sleep apnea. These findings suggest the importance of tailored extubation strategies and close perioperative monitoring in morbidly obese patients to mitigate extubation-related risks.

## Data Availability

The data used in this work are available upon reasonable request from the corresponding author.
